# Acute kidney injury: preclinical innovations, challenges, and opportunities for translation

**DOI:** 10.1186/s40697-015-0062-9

**Published:** 2015-09-01

**Authors:** Samuel A. Silver, Héloise Cardinal, Katelyn Colwell, Dylan Burger, Jeffrey G. Dickhout

**Affiliations:** Division of Nephrology, St. Michael’s Hospital, University of Toronto, Toronto, Canada; Division of Nephrology, Centre Hospitalier de l’Université de Montréal and CHUM research center, Montreal, Quebec Canada; Department of Medicine, Division of Nephrology, McMaster University and St. Joseph’s Healthcare Hamilton, Hamilton, Ontario Canada; Kidney Research Centre, Ottawa Hospital Research Institute, Department of Cellular and Molecular Medicine, University of Ottawa, Ottawa, Ontario Canada; Department of Medicine, Division of Nephrology, McMaster University and St. Joseph’s Healthcare Hamilton, 50 Charlton Avenue East, Hamilton, Ontario L8N 4A6 Canada

**Keywords:** Acute kidney injury, Endoplasmic reticulum stress, Translational research, Kidney transplant, Stem cells

## Abstract

**Background:**

Acute kidney injury (AKI) is a clinically important condition that has attracted a great deal of interest from the biomedical research community. However, acute kidney injury AKI research findings have yet to be translated into significant changes in clinical practice.

**Objective:**

This article reviews many of the preclinical innovations in acute kidney injury AKI treatment, and explores challenges and opportunities to translate these finding into clinical practice.

**Sources of Information:**

MEDLINE, ISI Web of Science

**Findings:**

This paper details areas in biomedical research where translation of pre-clinical findings into clinical trials is ongoing, or nearing a point where trial design is warranted. Further, the paper examines ways that best practice in the management of AKI can reach a broader proportion of the patient population experiencing this condition.

**Limitations:**

This review highlights pertinent literature from the perspective of the research interests of the authors for new translational work in AKI. As such, it does not represent a systematic review of all of the AKI literature.

**Implications:**

Translation of findings from biomedical research into AKI therapy presents several challenges. These may be partly overcome by targeting populations for interventional trials where the likelihood of AKI is very high, and readily predictable. Further, specific clinics to follow-up with patients after AKI events hold promise to provide best practice in care, and to translate therapies into treatment for the broadest possible patient populations.

## Abrégé

**Contexte:**

L’insuffisance rénale aiguë (IRA), état pathologique important du point de vue clinique, suscite beaucoup d’intérêt dans le milieu de la recherche biomédicale. On tarde toutefois à transposer les conclusions des recherches sur ce sujet en modifications substantielles dans la pratique clinique.

**Objectifs:**

Le présent article passe en revue nombre d’innovations précliniques dans le traitement de l’IRA et explore les défis que pose la transposition des conclusions dans la pratique clinique, ainsi que les occasions d’y parvenir.

**Conclusion:**

Le présent rapport expose en détail les domaines de la recherche biomédicale dans lesquels les conclusions précliniques sont actuellement transposées ou au point où des essais cliniques seront bientôt justifiés. De plus, le rapport examine des façons d’étendre le recours aux pratiques exemplaires dans la gestion de l’IRA à un plus grand nombre de patients atteints de cette pathologie.

**Limites de l’étude:**

La présente étude fait état de la littérature du point de vue des champs d'intérêt de recherche des auteurs pour le travail de transposition en IRA. Elle ne se veut toutefois pas un compte rendu exhaustif de la littérature scientifique sur l’IRA.

**Répercussions:**

La transposition des conclusions de la recherche biomédicale en traitement de l’IRA pose de nombreux défis. Ceux-ci peuvent être partiellement surmontés en effectuant des essais interventionnels sur des populations ciblées parmi lesquelles l’incidence d’IRA est très élevée et prévisible. De plus, le suivi par des cliniques spécifiques des patients à la suite d’un épisode d’IRA semble prometteur dans le cadre de l’adoption de pratiques exemplaires et de la transposition des thérapies en traitements pour le plus grand bassin de patients possible.

## Why is this report/review important?

Acute kidney injury is a common clinical condition, whose incidence is increasing. It is associated with both acute and chronic health implications. Unfortunately, few therapies for acute kidney injury exist, and it is challenging to translate new potential therapies to clinical practice.

## What are the key messages?

Valley 1 (bench to bedside) barriers may be overcome by targeting populations for interventional trials where the likelihood of acute kidney injury is very high and readily predictable (such as nephrotoxic drugs or delayed graft function). Valley 2 (research to practice) barriers may be overcome through more systematic follow-up of acute kidney injury patients to provide evidence-based treatments.

## Implications for future research/policy

There are several translational research opportunities in acute kidney injury to bridge the valley 1 and 2 barriers. This will require a transdisciplinary and patient-oriented approach to research and training, which is currently promoted through the Canadian Institutes of Health Research and the Kidney Research Scientist Core Education and National Training program.

## Introduction

Acute kidney injury (AKI) is an important healthcare issue worldwide. It affects 15-20 % of all hospital stays, with this figure reaching 30-40 % in patients admitted to critical care units [1, 2]. The incidence of AKI has increased over the last fifteen years, and is expected to double over the next decade [3]. AKI is associated with a significant increase in hospital length of stay and mortality [4]. Individuals who survive to leave the hospital after an episode of AKI are at persistent risk of adverse outcomes, including a 10-fold greater risk of chronic kidney disease (CKD), a 3-fold greater risk of end-stage renal disease (ESRD), and double the risk of premature death [5]. Despite substantial research efforts and resources dedicated to AKI, few interventions have impacted the prevention, extension, or recovery of this clinical syndrome [6].

**Fig. 1:**
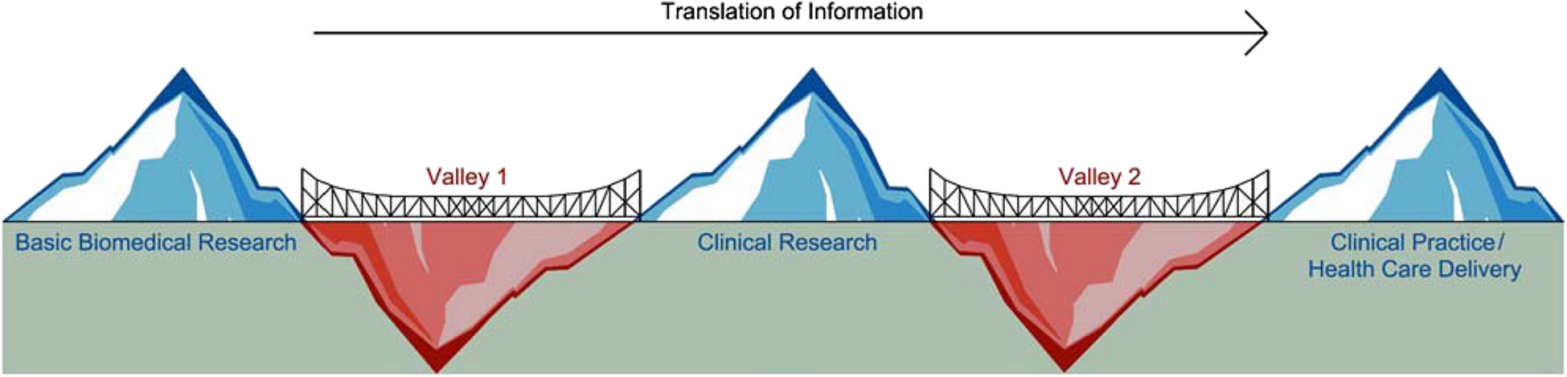
Bridging the "Death Valleys" of the Canadian healthcare landscape. Depiction of the barriers to putting research into practice in the Canadian healthcare landscape. In order to ensure that the system is sustainable and to enhance health outcomes, it is critical to bridge the gap between research and clinical practice. Valley 1 depicts the limited ability to translate information from basic biomedical research to clinical science and knowledge. Valley 2 depicts the inadequacy of the current healthcare system in synthesizing, disseminating and integrating research results into clinical practice and healthcare decision-making. To bridge the "Death Valleys" of the healthcare landscape, collective engagement in the strategy from all levels of government and the research community is necessary

The discrepancy between the new knowledge gained through biomedical and clinical research and actual improvements in patient care or health service organization for patients suffering from AKI illustrates a major challenge faced by the Canadian research community. Although Canada excels in biomedical research capacity and innovations, translation of these innovations into meaningful patient-based applications is a significant problem. Further, even when human applications and improvements in patient care are identified, the translation of this new knowledge into widespread clinical practice remains problematic [7]. This conundrum can be represented by a model depicting two "death valleys" of the biomedical research to clinical practice continuum (Fig. 1). A major funder of health-related research in Canada, the Canadian Institutes of Health Research (CIHR), argues for initiatives that will bring biomedical discoveries to the bedside (bridging valley 1) and translate findings from biomedical and clinical research into relevant healthcare decision making (bridging valley 2) [7]. The purpose of this review is to address some of the challenges and opportunities to bridge these two "death valleys" in the field of AKI research.

## Review

### New therapeutic targets for AKI treatment stemming from biomedical research: bridging valley 1

Over the last few decades, AKI has become the focus of extensive clinical and basic research efforts. Alongside the range of risk factors that predispose patients to AKI, the core pathology may be broken down into degenerative processes that affect the renal epithelium, vasculature, and innate and adaptive immune responses leading to worsening of this condition [8].

#### Inflammation

The pathophysiology of AKI includes nephron loss through tubular epithelial cell programmed cell death, or apoptosis and renal epithelial cell necrosis that trigger an immune response. These conditions lead to a cellular infiltrate resulting in a decline in the kidney’s filtration capacity [8]. Renal ischemia is one of the major causes of AKI. The pathogenesis of renal ischemia involves an acute inflammatory process leading to the increased expression of cytokines and chemokines [9]. Innate and adaptive immune cells participate in the renal ischemic inflammatory response, where T-regulatory cells (Tregs) play an important role in attenuating immunologic damage to the kidney by suppressing a tissue destructive inflammatory response to self-antigens [10]. These research findings have been slow to be adapted to clinical research since Tregs are difficult to isolate and multiply to provide enough cells for treatment [10]. However, in recent research efforts, it has been found that the interleukin (IL)-2/anti-IL-2 complex (IL-2C) mediates the preferential expansion of Tregs up to 4-fold *in vivo* [9]. This study used a mouse model of ischemia reperfusion injury (IRI), showed an improvement in renal function through a decrease in the rise of serum creatinine and blood urea nitrogen (BUN) by more than 50 %, with IL-2C administration. This was accompanied by an attenuation of renal injury score and apoptosis after IRI. IL-2C was also shown to increase tubular cell proliferation, and reduce renal fibrosis. As such, IL-2C-induced-Treg-expansion may be a viable option in clinical trials to decrease AKI and facilitate renal recovery.

#### Oxidative Stress

Mitochondrial dynamics are an important component of AKI. Alterations in mitochondrial function include fragmentation with reduction in adenosine triphosphate (ATP)-generating capacity, fission and subsequent apoptosis during the stress of ischemic injury, enhanced production of reactive oxygen species (ROS), and mitochondrial permeability transition-pore opening [11]. Mitochondrial dysfunction is further characterized by progressive accumulation of calcium and depression in oxidative phosphorylation [12]. Mitochondrial dysfunction leads to ROS generation that may mediate some pathological features of AKI due to acute tubular necrosis (ATN). Ischemia may lead to ROS production through mitochondrial dysfunction. To test if ROS scavenging directed at the mitochondria improved AKI outcome, the mitochondrial specific ROS scavenger, Mito-TEMPO, was used. Inulin-based measurements of glomerular filtration rate (GFR) fell to approximately 25 % of control in the cecal ligation puncture mouse model of sepsis-induced AKI [13]. When Mito-TEMPO was dosed at 10 mg/kg, GFR decline was limited to 50 %, and 96-hour survival was improved from 40 % to 80 % [13]. Another approach taken pre-clinically has been to stimulate mitochondrial biogenesis through Beta2-adrenergic receptor stimulation with formoterol. This approach improved renal function as shown by the normalization of serum creatinine levels to that of sham controls by 144 hours after IRI in a mouse model [14]. Thus, selectively improving mitochondrial function can reduce injury and ultimately reverse AKI. As formoterol is a Food and Drug Administration (FDA) approved therapeutic, safety trials in patients likely to experience AKI may be warranted, and extension of these trials to interventional randomized control trials would be advisable.

#### Endoplasmic Reticulum (ER) Stress

**Fig. 2:**
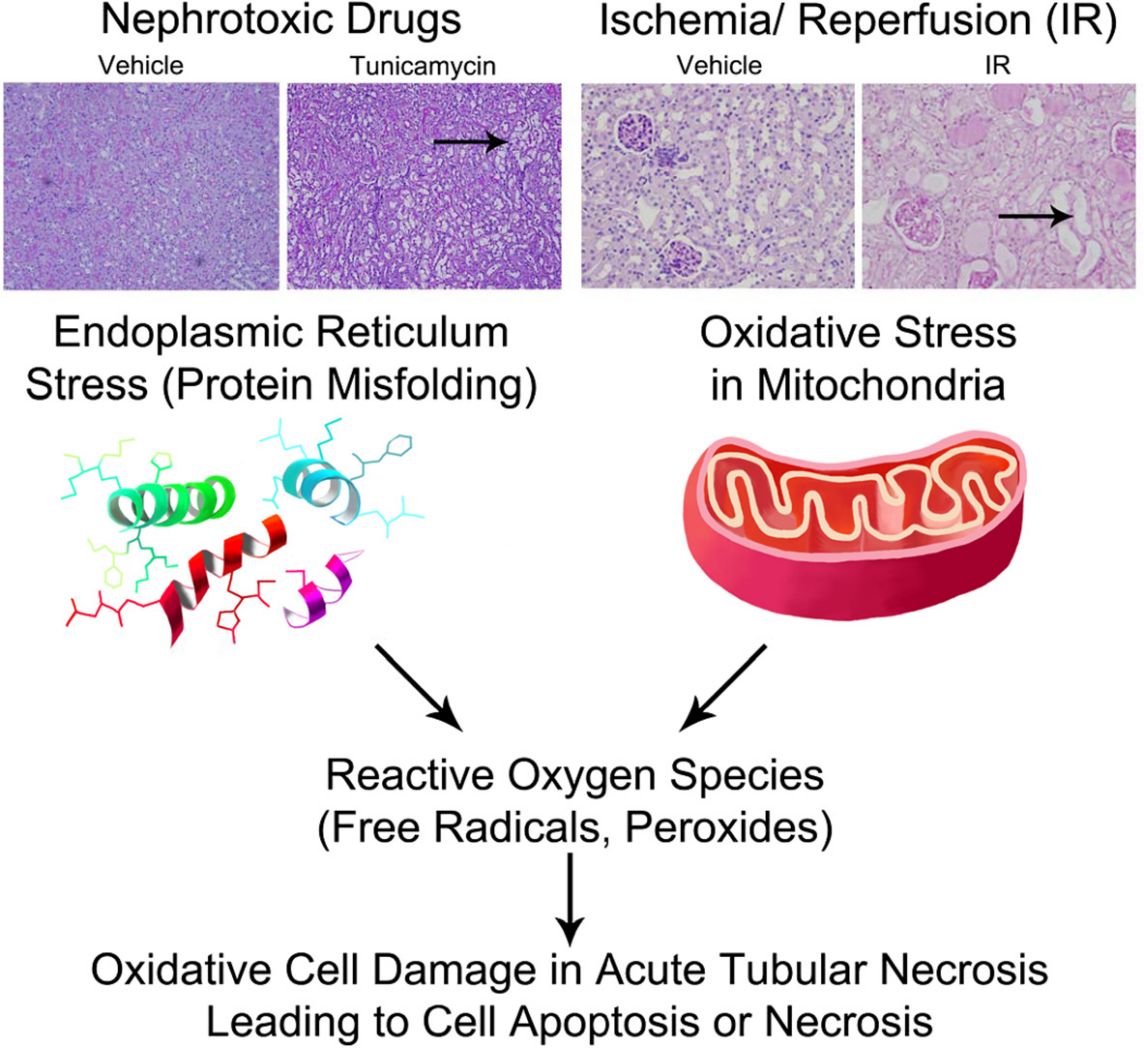
Acute kidney injury due to acute tubular necrosis. Acute tubular necrosis can be the result of nephrotoxins or ischemia to the kidney. Nephrotoxic drugs, such as tunicmycin, can induce ER stress caused by protein misfolding; while a lack of blood supply to the kidney can cause oxidative stress in the mitochondria. Both ER stress and oxidative stress have been shown to generate reactive oxygen species, ultimately leading to acute kidney injury

The process of ER stress has been linked to AKI from a variety of causes, such as ischemia, nephrotoxic drugs or contrast media [15–19]. ER stress is caused by the accumulation of misfolded proteins in the ER [19]. It has become clear that ER stress induction in the kidney generates AKI [19, 20]. The process of ER and oxidative stress leading to loss of renal function in AKI is summarized in Fig. 2. Diverse physiological and environmental stressors are also regulated through heat shock proteins (HSPs), which are molecular chaperones that are induced in response to cellular stresses that cause protein misfolding [21]. HSPs transiently bind to polypeptides to facilitate correct protein folding by preventing the aggregation of misfolded proteins. In rodent models of IRI-induced AKI, HSP induction was shown to provide protection against the increase in BUN and creatinine levels, preventing the increase in BUN from normal levels, and reducing the tubular necrosis and cast formation index from extensive to mild [22]. The beneficial effects of HSPs were time dependent, and function most efficiently when increased within 6 hours of the AKI-inducing insult. HSPs 70s and 90s are of particular importance in the regulation of protein folding, including the protein GRP78 [21]. ER stress-induced AKI has been shown to be associated with neutral lipid accumulation [23]. GRP78 overexpression reduces lipid accumulation generated by ER stress [23]. Low molecular weight chemical chaperones have been used to reduce ER stress and inhibit AKI due to nephrotoxins [20] and IRI [24].

Nephron epithelial cell loss during AKI results in part from apoptosis [25], and prolonged or severe ER stress increases expression of pro-apoptotic mediators including CHOP/GADD153 [26, 27]. It has been determined that the induction of ER stress in the kidney results in CHOP/GADD153 upregulation and ATN, which is strongly associated with the occurrence of apoptotic cells in the region of injury. In a mouse model of nephrotoxin-induced AKI, the low molecular weight chemical chaperone, 4-phenylbutyrate (4-PBA), reduced tubular injury score by approximately half [20]. This effect was accompanied by a reduction in the expression of the CHOP/GADD153 protein both in the animal model as well as in human proximal tubular epithelial cells. Detailed molecular analysis in the human proximal tubular cell model revealed the direct effect of the CHOP/GADD153 protein in inducing tubular cell death. Return to the mouse model, where genetic disruption of the CHOP/GADD153 protein was performed, showed protection of the kidney from AKI by both routine pathological assessment, and ultrastructural analysis. In this study, the move from pre-clinical animal models to human cell systems illustrates the next step in translation in biomedical sciences in preparation for first in human trials. Thus, the induction of ER stress by various pathophysiological mediators may contribute to AKI through tubular epithelial cell death. Drugs that inhibit ER stress, such as the molecular chaperone 4-PBA, have demonstrated efficacy in reducing AKI in preclinical studies [20] and have satisfactory patient safety profiles in patients with liver cirrhosis and cystic fibrosis [28, 29]. However, no safety profiles exist for renal patients. Use of these chaperones may represent the next step in translation from biomedical research to clinical trials in AKI. It is likely that chemical chaperone therapy would be most efficacious if given prophylactically in patients at high risk of developing AKI.

#### Autoimmunity

Microvascular injury and endothelial dysfunction have recently emerged as pivotal elements in the pathogenesis of AKI [8, 30]. Following IRI, endothelial dysfunction/injury and apoptosis further compromise microcirculatory renal blood flow through decreased vasodilatory capacity [31], coagulation activation and the formation of microvascular thrombi [31], and increased rolling/adhesion of inflammatory cells [8]. Because the regenerative capacity of endothelial cells in peritubular capillaries (PTC) appears limited [32], AKI-related endothelial injury and apoptosis lead to PTC rarefaction [32, 33], interstitial fibrosis, and increase the risk of CKD [34].

Innate immunity, in particular complement activation, mediates IRI and AKI after kidney transplantation [35]. The concept of ‘innate autoimmunity’ as a participating factor in IRI has been put forward by Zhang et al. [36]. In murine studies, periods of ischemic stress/hypoxia induced alterations in surface epitopes in the intestines and skeletal muscles. Binding of a naturally-occurring IgM antibody to this self-antigen (nonmuscle myosin heavy chain) activated complement and caused tissue injury [36]. These data suggest that autoimmunity could accelerate tissue injury due to IRI.

Apoptotic endothelial cells release a C-terminal fragment of perlecan that was named LG3 because of its 3 laminin G motifs [37]. A circulating autoantibody to LG3, anti-LG3, is elevated pre- and post-transplant in kidney transplant recipients who experience acute vascular rejection [38]. In mice, passive transfer of anti-LG3 antibodies increases vascular inflammation and complement deposition in aortic vascular allografts when the allograft is made ischemic prior to transplantation [38]. This suggests that anti-LG3 autoantibodies can enhance the alloimmune response in the presence of ischemic stress, which may create permissive conditions through the local expression of LG3 in the vascular wall. Because microvascular injury is a prominent factor in IRI [8], Hamelin et al. speculated that pre-transplant anti-LG3 autoantibodies might increase the risk and severity of delayed graft function (DGF), and their recent preliminary studies support this hypothesis [39]. If this finding is confirmed in larger studies, therapeutic modalities such as intravenous gammaglobulins or plasmapheresis could eventually be tested to prevent DGF in patients with elevated anti-LG3 levels.

#### Regenerative medicine and AKI

One of the main challenges for new clinical therapies in AKI has been the heterogeneous nature and diverse causes of the disease [8]. The administration of stem/progenitor cells offers an alternative approach to the targeting of specific pathophysiological processes. As highly proliferative cells capable of differentiation into multiple lineages, progenitor cells have the theoretical ability to travel to the site of injury and transdifferentiate into healthy, functional tissue [40]. There have been numerous studies in animals reporting restoration of function post-AKI following administration of cells with beneficial effects including reduced inflammation, accelerated tubular regeneration, promotion of angiogenesis, and inhibition of apoptosis/necrosis [40–42].

By far the most widely and consistently employed cell population for the treatment of AKI is mesenchymal stromal cells (MSCs), which are heterogeneous, rare cells that may be found in bone marrow, peripheral blood, adipose tissue, skeletal muscle, umbilical cord wall/blood, and amniotic fluid [41]. A recent meta-analysis of animal studies of MSC therapy in AKI showed consistent reduction in serum creatinine across multiple injury models (ischemic, nephrotoxic), supporting their protective effects [43]. In addition to MSCs, beneficial effects have been reported in animals treated with hematopoietic stem cells [44], induced pluripotent stem cells [45], endothelial progenitor cells [46], and mature endothelial cells [47]. In contrast to these studies, Burger et al. and others have shown that certain progenitor populations increase injury in experimental AKI, thereby highlighting the critical need for careful selection of cells to ensure safe promotion of recovery [48–50].

A number of ongoing clinical trials are aimed at transitioning this experimental therapy into clinical use (transition through valley 1). One study (NCT01602328-clinicaltrials.gov) is examining effects of a MSC-based therapy (AC607) in AKI after cardiac surgery, while another (NCT01275612) is assessing MSCs in cisplatin-induced AKI. In addition, several studies are examining the effects of MSCs in kidney transplantation (NCT01429038; NCT00752479; NCT00658073; NCT00734396). A recently completed phase one study of MSCs for the treatment of acute allograft rejection after renal transplantation suggested clinical feasibility and safety [51].

Interestingly, the majority of preclinical studies in cell therapy have reported exceptionally low levels of cell engraftment and limited trans-differentiation into damaged tissue [52, 53]. Such limited engraftment suggests that infused cells act in a paracrine fashion to achieve their effects. Consistent with this, conditioned media from MSCs has been shown to provide benefit in AKI [54, 55]. While there has been substantial interest in the role of secreted factors released by infused cells, extracellular vesicles may also be important. Extracellular vesicles are membrane-derived vesicles that are released from cells into the extracellular space and are increasingly recognized as mediators of cell-cell communication [56]. While there is not presently a consensus terminology, major classes include exosomes (40–100 nm in size), microparticles (100–1000 nm in size) and apoptotic bodies (1–4 um in size) [56]. Several studies have reported that administration of extracellular vesicles derived from progenitor populations may be beneficial in AKI. Putative mechanisms of action include the transfer of micro-RNA (miRNA) to injured cells [57, 58]. It is noteworthy that the majority of the above studies have examined mixed populations of extracellular vesicles, which contain both exosomes and microparticles, and the relative contributions of individual vesicle subpopulations are unclear.

The use of extracellular vesicles may offer several advantages over whole cell therapy, which may aid in the translation into clinical therapy. For example, due to their small size, vesicles may be more likely to reach the site of injury compared with cells, which can become trapped in microvascular beds [59]. The relatively small size of vesicles might thereby eliminate the need for direct tissue delivery to optimize therapeutic efficacy, a condition that may be required with whole cells. Vesicles lack capacity to proliferate, thus reducing the theoretical risk of spontaneous tumour development post-administration. Finally, certain vesicle subpopulations (i.e. exosomes) may have decreased immunogenicity compared with their cells of origin [60, 61].

**Fig. 3:**
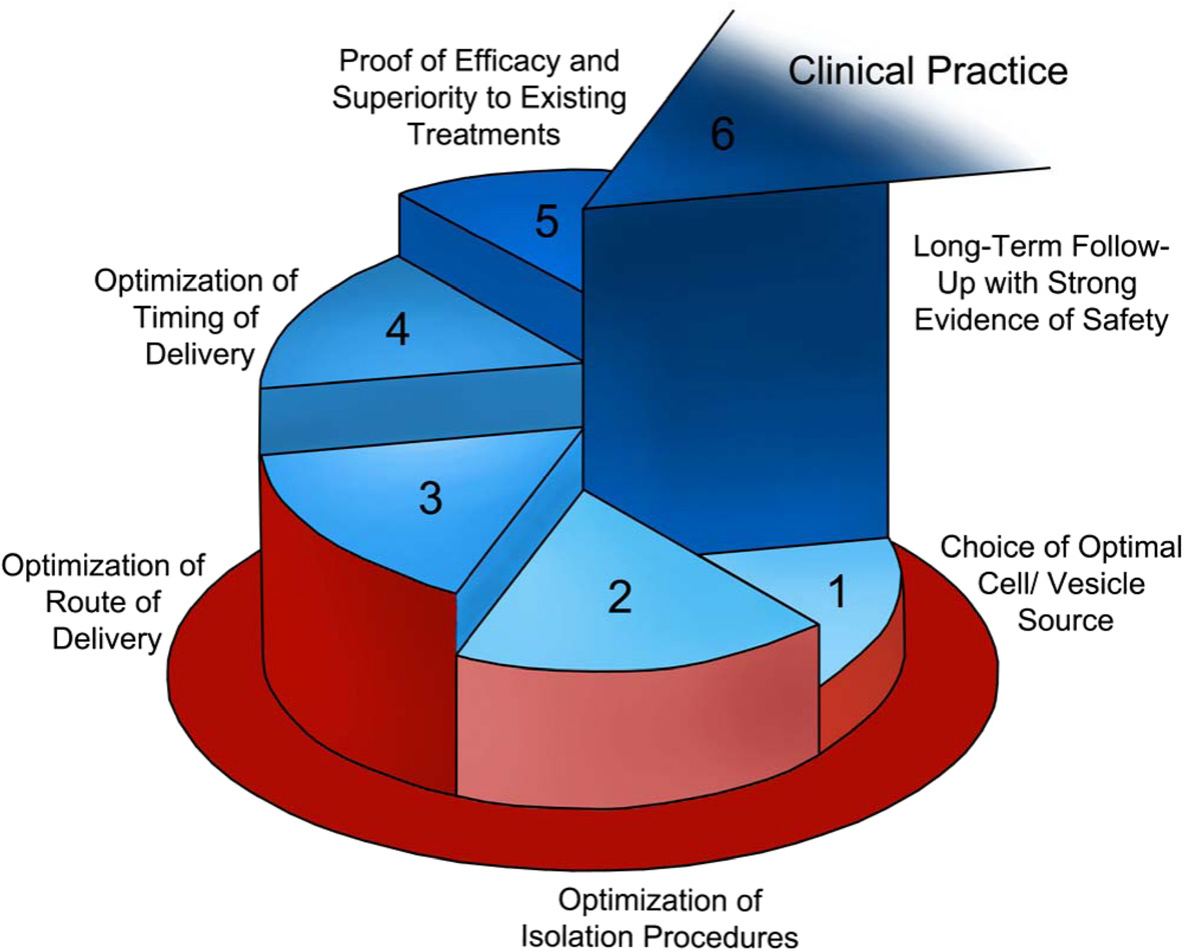
Steps to translate regenerative therapy for acute kidney injury into clinical practice. Cell-based therapy faces a number of unique barriers to be overcome in order to translate research into clinical practice. Firstly, the most effective population of cells to use for therapy is unclear. While beneficial effects have been reported from various cell populations, there is a need for comparisons of efficacy across different cell types. Second, the optimum cell isolation procedure has yet to be identified. Next, the optimum route of cell delivery and timing of delivery in order to promote recovery is unknown. In order to bring acute kidney injury therapy to a new level through regenerative medicine, effectiveness of cell-based treatments must be proven superior to treatment methods currently in use. The final step addresses long-term follow-up with subjects to ensure safety of cell based therapy

Cell therapy has shown therapeutic promise in preclinical studies that, if realized clinically, would be transformative within the field of AKI. Nevertheless, uptake of this preclinical innovation has been slow and while the benefits of cell therapy have been established for over a decade, clinical trials are only recently established. While this is likely due in part to a greater burden for regulatory approval, preclinical research has also failed to address several key steps in the optimization of cell-based therapy. First, the most effective population of cells for promoting recovery has not been established through direct comparison of various cell populations. Secondly, isolation procedures have not been standardized and the most appropriate source (i.e. allogeneic vs autologous; peripheral blood vs umbilical cord blood) has not been established. Third, the most effective route and timing of delivery is unclear. Finally, questions of long-term safety have not been adequately addressed, as long-term follow-up has only rarely been done in animal models. Collectively, the failure to address these key steps has led to a paucity of information essential for appropriate trial design, without which therapeutic development is impossible. By contrast, preclinical work examining extracellular vesicles is at a much earlier stage. Because of this, preclinical research has the opportunity to better inform potential clinical studies on extracellular vesicles in AKI through optimization of therapy. Such information would lead to improved design of clinical trials, and a more rapid translation of results through valleys 1 and 2 into clinical practice (see Fig. 3).

### Promising opportunities for translation of clinical research findings to everyday clinical practice and service delivery in AKI: bridging valley 2

As mentioned above, the development of novel therapeutic strategies to prevent or treat AKI based on findings from basic science has been slow, partly because of the multifactorial pathophysiology of AKI. However, challenges in the prediction and early identification of this condition [62] have also contributed to the limited success of therapeutic innovations in AKI. Elevations in serum creatinine remain the most common method for diagnosis of AKI, even though kidney damage precedes increases in creatinine. Creatinine also cannot differentiate between the multiple causes of AKI. This creates a situation where potential therapies can only be tested in patients with established AKI, where they may be less effective. To address this challenge, a number of serum and urinary biomarkers have been evaluated in order to diagnose AKI at an earlier stage than serum creatinine. A discussion of these biomarkers and their strengths and limitations has recently been reviewed [62]. Most are still available only in the context of research studies, but the FDA has approved the NephroCheck® test that combines the urinary biomarkers insulin-like growth factor binding protein 7 and tissue inhibitor of metalloproteinases 2 [63, 64]. NephroCheck® has only been validated in critically ill patients and has yet to demonstrate an impact on patient outcomes. Therefore more work remains to determine its exact role in the diagnosis and treatment of AKI.

Despite these challenges, there are several opportunities in AKI research to bridge valley 2. These include, AKI after kidney transplantation and standardizing follow-up care for AKI survivors to improve long-term patient outcomes.

#### Post-transplant AKI

Kidney transplantation is an excellent clinical setting for translational research on the prevention of AKI, since the timing of IRI is precisely known. In the immediate post-transplant period, IRI to the transplanted organ leads to AKI in 20 %-50 % of transplantations from deceased donors [65–67]. Post-transplant AKI, or DGF, is associated with an increased risk of acute rejection [68, 69] and reduced long-term graft survival [68, 70, 71].

Predicting which recipient will develop DGF is important to provide information to patients and physicians on the expected post-transplant evolution, and may influence the selection of induction therapy [72]. In order to facilitate prediction of DGF, user-friendly tools such as nomograms [73, 74] have been developed and validated in kidney transplant patients [75–77]. Web-based risk calculators for DGF also exist [76, 78], including applications that can be downloaded on smartphones or tablets [78]. The availability of these web-based tools is an excellent example of translation of clinical research to everyday decision–making. Nevertheless, the predictive nomograms are not routinely used in clinical practice. This may be due to their perceived limited clinical applicability [79]. As immunosuppressive or allocation strategies that differ according to nomogram scores have not been tested, physicians may not feel comfortable modifying their current treatment schemes on the basis of these scores [79]. This is especially relevant since reported diagnostic accuracies for the existing models are reasonable but not strong, with C-statistics of 0.71-0.73 [76, 78]. To bridge valley 2, future studies will need to test the clinical usefulness of the scores for selecting different therapeutic strategies.

One unique aspect of AKI prevention in kidney transplantation is the possibility of managing the donor and/or the organ before implantation. In the last decade, an example of successful translation of basic/clinical research to clinical practice has been the use of hypothermic machine perfusion instead of static cold storage to preserve the kidney allograft between procurement and implantation [80]. A recent meta-analysis shows that use of hypothermic machine perfusion leads to a relative risk for DGF of 0.81 when compared to cold storage [81], a figure that increases to 40-45 % relative risk reductions for DGF in recipients of extended criteria donors and of donors after cardiac arrest [82, 83]. Despite proven benefit in the prevention of DGF, machine perfusion is not being used uniformly across Canada. Although little data exists on the factors that limit its use, the latter may include uncertainty about the cost-effectiveness of machine perfusion [84], machine availability and cost, or experience and interest of the surgical team harvesting the organs. The importance of knowledge translation, or raising knowledge users’ awareness of research findings and facilitating the use of those findings [85], is increasingly being recognized [86]. Research including all important stakeholders to gather data on the frequency of machine perfusion, the factors that limit its use, and potential solutions to overcome these barriers is now needed to truly change everyday clinical practice [86].

#### Long-term AKI outcomes

One of the barriers to optimal patient care has been that therapeutic AKI research often focuses on short-term outcomes (such as 90-day mortality) [87, 88], ignoring the kidney and cardiovascular morbidity that affects AKI survivors. This gap in knowledge creation may have resulted in a missed opportunity to improve long-term outcomes for patients who survive AKI [89]. Without a standard model for post-AKI care, it is not surprising that only 40 % of patients who required dialysis for AKI and recovered sufficient kidney function to stop dialysis saw a nephrologist within 90-days of hospital discharge [90]. This represents an important care gap since a nephrologist visit within 90-days of discharge has been associated with a 25 % relative mortality reduction compared to patients who do not see a nephrologist after hospital discharge [90].

In order to more effectively translate evidence-based recommendations to AKI survivors, an approach taken by two tertiary hospitals in Toronto, Canada has been the establishment of an AKI Follow-up Clinic. All hospitalized patients with a serum creatinine that at least doubled compared to baseline or who received dialysis for AKI are potentially eligible for the AKI Follow-up Clinic if they survive to hospital discharge. Clinic appointments are arranged within 30-days of discharge, but up to 90-days is acceptable. Visits consist of a standardized assessment that highlights blood pressure and urine albumin control, review of quarterly blood work, cardiovascular risk reduction, and management of kidney disease complications. Evidence-based guidelines are also provided on the standardized assessment templates for treatment of CKD [91], hypertension [92], hyperlipidemia [93, 94], and diabetic nephropathy [95].

While the experiences of the AKI Follow-up Clinic are preliminary, this intervention offers several opportunities to translate clinical knowledge into practice. First, AKI survivor follow-up may present an opportunity to expose previously unrecognized CKD and establish nephrology contact [3]. CKD is the most important risk factor for AKI [96], and a previous cohort demonstrated that 60 % of AKI survivors have pre-existing CKD and have never seen a nephrologist [97]. Improving healthcare access for this population may address some of the barriers associated with suboptimal dialysis initiation (defined as dialysis initiation in-hospital and/or temporary vascular access) [98, 99], thereby reducing healthcare costs and patient morbidity [100, 101].

Second, the AKI Follow-up Clinic may allow for more appropriate treatment of CKD and cardiovascular complications, since studies suggest that nephrologists are skilled at recognizing and managing such complications according to evidence-based guidelines [102, 103]. Potential opportunities for valley 2 translation include the treatment of hypertension, proteinuria, and hyperlipidemia. This is especially important for hospitalized AKI survivors, since up to 67 % of patients admitted to hospital have unintended medication discrepancies at discharge [104, 105]. These discrepancies have been associated with hospital readmission and death, particularly for cessation of chronic disease medications [106]. Thus, an AKI Follow-up Clinic may help maximize the appropriate use of anti-platelet agents, renin-angiotensin inhibitors, and statins.

Third, the AKI Follow-up Clinic provides nephrologists with an opportunity to educate primary care providers and specialists on AKI and its downstream complications. It is only now being appreciated that AKI survivors have similar long-term outcomes as patients with diabetes and survivors of a ST-elevation myocardial infarction [107, 108]. Adverse outcomes seem to be attenuated, but still persist in patients whose serum creatinine approaches its pre-hospital baseline within 90 days of discharge [109]. Therefore, some experts have suggested that an “episode of AKI” be documented in a patient’s past medical history [3]. Incorporating this recommendation into daily practice will require effective knowledge translation and education strategies, which an AKI Follow-up Clinic is well-positioned to accomplish through dictated clinic letters.

Despite the theoretical benefits of the AKI Follow-up Clinic, several challenges to successful translation and implementation remain. First, the reasons for AKI survivor non-referral are unknown. This will require broad stakeholder engagement with healthcare providers, patients, caregivers, and administrators. Qualitative and quality improvement methods will be required to design effective and sustainable AKI survivor referral pathways [110]. Second, there is no high quality evidence to prove that the processes implemented in the AKI Follow-up Clinic will reduce the morbidity or mortality among AKI survivors. This will require observational and interventional studies to support the potential mechanisms offered above.

If successful in establishing an AKI survivor care pathway, this clinic could help facilitate translational research by bringing together basic science and clinical researchers to study the mechanisms of AKI to CKD transition in a well-defined population. Potential areas of investigation include the role of fibrosis in AKI [111], biomarkers of CKD progression [112], and bedside tools to predict adverse events post-AKI [113, 114]. Such interdisciplinary collaborations via specialty clinics have been successful in other disciplines [115], and may also help nephrology to translate research findings into health benefits for patients.

## Conclusion

In this paper, we have shown examples of successful translational research, areas where improvement is urgently needed, and new opportunities for improving the care of AKI. For the last decade, the Canadian nephrology research community has responded to the need for translational research by creating training and research networks promoting a transdisciplinary approach, such as the Kidney Research Scientist Core Education and National Training (KRESCENT) program, the Canadian National Transplant Research Program (CNTRP), and the Canadian Kidney Knowledge Translation and Generation Network (www.CANN-NET.ca). CIHR’s strategy for patient oriented research is now creating a unique opportunity for Canada to bring together research professionals from various orientations and create a nation-wide, integrated research network in the field of nephrology. In the future, this network will improve Canada’s success in bridging the two valleys of the continuum in health research.

## Abbreviations

AKI: Acute kidney injury
BUN: blood urea nitrogen
CKD: chronic kidney disease
DGF: delayed graft function
ESRD: end-stage renal disease
FDA: Food and Drug Administration
CIHR: Canadian Institutes of Health Research
Tregs: T-regulatory cells
IL-2C: IL-2/anti-IL-2 complex
IRI: ischemia reperfusion injury
ROS: reactive oxygen species
ATN: acute tubular necrosis
ER: Endoplasmic Reticulum
HSPs: heat shock proteins
PTC: peritubular capillaries
MSCs: mesenchymal stromal cells
miRNA: micro-RNA
DGF: delayed graft function
KRESCENT: Kidney Research Scientist Core Education and National Training
CNTRP: Canadian National Transplant Research Program

## References

[1] Susantitaphong P, Cruz DN, Cerda J, Abulfaraj M, Alqahtani F, Koulouridis I (2013). World incidence of AKI: a meta-analysis. Clin J Am Soc Nephrol.

[2] Bellomo R, Kellum JA, Ronco C (2012). Acute kidney injury. Lancet.

[3] Goldstein SL, Jaber BL, Faubel S, Chawla LS, Acute Kidney Injury Advisory Group of American Society of Nephrology (2013). AKI transition of care: a potential opportunity to detect and prevent CKD. Clin J Am Soc Nephrol.

[4] Chertow GM, Burdick E, Honour M, Bonventre JV, Bates DW (2005). Acute kidney injury, mortality, length of stay, and costs in hospitalized patients. J Am Soc Nephrol.

[5] Coca SG, Singanamala S, Parikh CR (2012). Chronic kidney disease after acute kidney injury: a systematic review and meta-analysis. Kidney Int.

[6] Siew ED, Himmelfarb J (2013). The inexorable rise of AKI: can we bend the growth curve?. J Am Soc Nephrol.

[7] Canadian Institute of Health Research. Strategy for Patient-Oriented Research. August 2011. Available from: http://www.cihr-irsc.gc.ca/e/documents/P-O_Research_Strategy-eng.pdf.

[8] Molitoris BA (2014). Therapeutic translation in acute kidney injury: the epithelial/endothelial axis. J Clin Invest.

[9] Kim MG, Koo TY, Yan JJ, Lee E, Han KH, Jeong JC (2013). IL-2/anti-IL-2 complex attenuates renal ischemia-reperfusion injury through expansion of regulatory T cells. J Am Soc Nephrol.

[10] Wang YM, Alexander SI (2013). IL-2/anti-IL-2 complex: a novel strategy of in vivo regulatory T cell expansion in renal injury. J Am Soc Nephrol.

[11] Brooks C, Wei Q, Cho SG, Dong Z (2009). Regulation of mitochondrial dynamics in acute kidney injury in cell culture and rodent models. J Clin Invest.

[12] Tabara LC, Poveda J, Martin-Cleary C, Selgas R, Ortiz A, Sanchez-Nino MD (2014). Mitochondria-targeted therapies for acute kidney injury. Expert Rev Mol Med.

[13] Patil NK, Parajuli N, MacMillan-Crow LA, Mayeux PR (2014). Inactivation of renal mitochondrial respiratory complexes and manganese superoxide dismutase during sepsis: mitochondria-targeted antioxidant mitigates injury. Am J Physiol Renal Physiol.

[14] Jesinkey SR, Funk JA, Stallons LJ, Wills LP, Megyesi JK, Beeson CC (2014). Formoterol restores mitochondrial and renal function after ischemia-reperfusion injury. J Am Soc Nephrol.

[15] Prachasilchai W, Sonoda H, Yokota-Ikeda N, Ito K, Kudo T, Imaizumi K (2009). The protective effect of a newly developed molecular chaperone-inducer against mouse ischemic acute kidney injury. J Pharmacol Sci.

[16] Prachasilchai W, Sonoda H, Yokota-Ikeda N, Oshikawa S, Aikawa C, Uchida K (2008). A protective role of unfolded protein response in mouse ischemic acute kidney injury. Eur J Pharmacol.

[17] Peyrou M, Hanna PE, Cribb AE (2007). Cisplatin, gentamicin, and p-aminophenol induce markers of endoplasmic reticulum stress in the rat kidneys. Toxicol Sci.

[18] Peyrou M, Cribb AE (2007). Effect of endoplasmic reticulum stress preconditioning on cytotoxicity of clinically relevant nephrotoxins in renal cell lines. Toxicol In Vitro.

[19] Dickhout JG, Krepinsky JC (2009). Endoplasmic reticulum stress and renal disease. Antioxid Redox Signal.

[20] Carlisle RE, Brimble E, Werner KE, Cruz GL, Ask K, Ingram AJ (2014). 4-Phenylbutyrate Inhibits Tunicamycin-Induced Acute Kidney Injury via CHOP/GADD153 Repression. PLoS ONE.

[21] Dickhout JG, Carlisle RE, Austin RC (2011). Inter-Relationship between Cardiac Hypertrophy, Heart Failure and Chronic Kidney Disease – Endoplasmic Reticulum Stress as a Mediator of Pathogenesis. Circ Res.

[22] Kelly KJ, Baird NR, Greene AL (2001). Induction of stress response proteins and experimental renal ischemia/reperfusion. Kidney Int.

[23] Lhotak S, Sood S, Brimble E, Carlisle RE, Colgan SM, Mazzetti A (2012). ER stress contributes to renal proximal tubule injury by increasing SREBP-2-mediated lipid accumulation and apoptotic cell death. Am J Physiol Renal Physiol.

[24] Gao X, Fu L, Xiao M, Xu C, Sun L, Zhang T (2012). The nephroprotective effect of tauroursodeoxycholic acid on ischaemia/reperfusion-induced acute kidney injury by inhibiting endoplasmic reticulum stress. Basic Clin Pharmacol Toxicol.

[25] Sharfuddin AA, Molitoris BA (2011). Pathophysiology of ischemic acute kidney injury. Nat Rev Nephrol.

[26] Wu X, He Y, Jing Y, Li K, Zhang J (2010). Albumin overload induces apoptosis in renal tubular epithelial cells through a CHOP-dependent pathway. OMICS.

[27] Kimura K, Jin H, Ogawa M, Aoe T (2008). Dysfunction of the ER chaperone BiP accelerates the renal tubular injury. Biochem Biophys Res Commun.

[28] Rockey DC, Vierling JM, Mantry P, Ghabril M, Brown RS, Alexeeva O (2014). Randomized, double-blind, controlled study of glycerol phenylbutyrate in hepatic encephalopathy. Hepatology.

[29] Zeitlin PL, Diener-West M, Rubenstein RC, Boyle MP, Lee CK, Brass-Ernst L (2002). Evidence of CFTR function in cystic fibrosis after systemic administration of 4-phenylbutyrate. Mol Ther.

[30] Basile DP, Yoder MC (2014). Renal endothelial dysfunction in acute kidney ischemia reperfusion injury. Cardiovasc Hematol Disord Drug Targets.

[31] Goligorsky MS, Brodsky SV, Noiri E (2004). NO bioavailability, endothelial dysfunction, and acute renal failure: new insights into pathophysiology. Semin Nephrol.

[32] Basile DP, Friedrich JL, Spahic J, Knipe N, Mang H, Leonard EC (2011). Impaired endothelial proliferation and mesenchymal transition contribute to vascular rarefaction following acute kidney injury. Am J Physiol Renal Physiol.

[33] Kwon O, Hong SM, Sutton TA, Temm CJ (2008). Preservation of peritubular capillary endothelial integrity and increasing pericytes may be critical to recovery from postischemic acute kidney injury. Am J Physiol Renal Physiol.

[34] Basile DP (2007). The endothelial cell in ischemic acute kidney injury: implications for acute and chronic function. Kidney Int.

[35] Ponticelli C (2014). Ischaemia-reperfusion injury: a major protagonist in kidney transplantation. Nephrol Dial Transplant.

[36] Zhang M, Alicot EM, Chiu I, Li J, Verna N, Vorup-Jensen T (2006). Identification of the target self-antigens in reperfusion injury. J Exp Med.

[37] Raymond MA, Desormeaux A, Laplante P, Vigneault N, Filep JG, Landry K (2004). Apoptosis of endothelial cells triggers a caspase-dependent anti-apoptotic paracrine loop active on VSMC. FASEB J.

[38] Cardinal H, Dieude M, Brassard N, Qi S, Patey N, Soulez M (2013). Antiperlecan antibodies are novel accelerators of immune-mediated vascular injury. Am J Transplant.

[39] Hamelin, K, H-RM, S Morissette, MH Hébert, H Cardinal. Pre-Transplant Anti-LG3 Antibodies Enhance Allograft Dysfunction in Kidney Transplant Recipients with Delayed or Slow Graft Function*.* American Transplant Congress (abstract). 2015.

[40] Togel FE, Westenfelder C (2012). Kidney protection and regeneration following acute injury: progress through stem cell therapy. Am J Kidney Dis.

[41] Morigi M, Benigni A (2013). Mesenchymal stem cells and kidney repair. Nephrol Dial Transplant.

[42] Patschan D, Patschan S, Muller GA (2011). Endothelial progenitor cells in acute ischemic kidney injury: strategies for increasing the cells' renoprotective competence. Int J Nephrol.

[43] Wang Y, He J, Pei X, Zhao W (2013). Systematic review and meta-analysis of mesenchymal stem/stromal cells therapy for impaired renal function in small animal models. Nephrology (Carlton).

[44] Li B, Cohen A, Hudson TE, Motlagh D, Amrani DL, Duffield JS (2010). Mobilized human hematopoietic stem/progenitor cells promote kidney repair after ischemia/reperfusion injury. Circulation.

[45] Lee PY, Chien Y, Chiou GY, Lin CH, Chiou CH, Tarng DC (2012). Induced pluripotent stem cells without c-Myc attenuate acute kidney injury via downregulating the signaling of oxidative stress and inflammation in ischemia-reperfusion rats. Cell Transplant.

[46] Patschan D, Patschan S, Wessels JT, Becker JU, David S, Henze E (2010). Epac-1 activator 8-O-cAMP augments renoprotective effects of syngeneic [corrected] murine EPCs in acute ischemic kidney injury. Am J Physiol Renal Physiol.

[47] Brodsky SV, Yamamoto T, Tada T, Kim B, Chen J, Kajiya F (2002). Endothelial dysfunction in ischemic acute renal failure: rescue by transplanted endothelial cells. Am J Physiol Renal Physiol.

[48] Burger D, Gutsol A, Carter A, Allan DS, Touyz RM, Burns KD (2012). Human cord blood CD133+ cells exacerbate ischemic acute kidney injury in mice. Nephrol Dial Transplant.

[49] Patschan D, Backhaus R, Elle HJ, Schwarze K, Henze E, Becker JU (2013). Angiopoietin-2 modulates eEOC-mediated renoprotection in AKI in a dose-dependent manner. J Nephrol.

[50] Gheisari Y, Ahmadbeigi N, Aghaee-Bakhtiari SH, Nassiri SM, Amanpour S, Azadmanesh K (2013). Human unrestricted somatic stem cell administration fails to protect nude mice from cisplatin-induced acute kidney injury. Nephron Exp Nephrol.

[51] Reinders ME, de Fijter JW, Roelofs H, Bajema IM, de Vries DK, Schaapherder AF (2013). Autologous bone marrow-derived mesenchymal stromal cells for the treatment of allograft rejection after renal transplantation: results of a phase I study. Stem Cells Transl Med.

[52] Kean TJ, Lin P, Caplan AI, Dennis JE (2013). MSCs: Delivery Routes and Engraftment, Cell-Targeting Strategies, and Immune Modulation. Stem Cells Int.

[53] Morigi M, De Coppi P (2014). Cell therapy for kidney injury: different options and mechanisms--mesenchymal and amniotic fluid stem cells. Nephron Exp Nephrol.

[54] Bi B, Schmitt R, Israilova M, Nishio H, Cantley LG (2007). Stromal cells protect against acute tubular injury via an endocrine effect. J Am Soc Nephrol.

[55] Zarjou A, Kim J, Traylor AM, Sanders PW, Balla J, Agarwal A (2011). Paracrine effects of mesenchymal stem cells in cisplatin-induced renal injury require heme oxygenase-1. Am J Physiol Renal Physiol.

[56] Burger D, Schock S, Thompson CS, Montezano AC, Hakim AM, Touyz RM (2013). Microparticles: biomarkers and beyond. Clin Sci (Lond).

[57] Cantaluppi V, Gatti S, Medica D, Figliolini F, Bruno S, Deregibus MC (2012). Microvesicles derived from endothelial progenitor cells protect the kidney from ischemia-reperfusion injury by microRNA-dependent reprogramming of resident renal cells. Kidney Int.

[58] de Almeida DC, Donizetti-Oliveira C, Barbosa-Costa P, Origassa CS, Camara NO (2013). In search of mechanisms associated with mesenchymal stem cell-based therapies for acute kidney injury. Clin Biochem Rev.

[59] Schrepfer S, Deuse T, Reichenspurner H, Fischbein MP, Robbins RC, Pelletier MP (2007). Stem cell transplantation: the lung barrier. Transplant Proc.

[60] Frangsmyr L, Baranov V, Nagaeva O, Stendahl U, Kjellberg L, Mincheva-Nilsson L (2005). Cytoplasmic microvesicular form of Fas ligand in human early placenta: switching the tissue immune privilege hypothesis from cellular to vesicular level. Mol Hum Reprod.

[61] Ichim TE, Zhong Z, Kaushal S, Zheng X, Ren X, Hao X (2008). Exosomes as a tumor immune escape mechanism: possible therapeutic implications. J Transl Med.

[62] Alge JL, Arthur JM (2015). Biomarkers of AKI: a review of mechanistic relevance and potential therapeutic implications. Clin J Am Soc Nephrol.

[63] Bihorac A, Chawla LS, Shaw AD, Al-Khafaji A, Davison DL, Demuth GE (2014). Validation of cell-cycle arrest biomarkers for acute kidney injury using clinical adjudication. Am J Respir Crit Care Med.

[64] Kashani K, Al-Khafaji A, Ardiles T, Artigas A, Bagshaw SM, Bell M (2013). Discovery and validation of cell cycle arrest biomarkers in human acute kidney injury. Crit Care.

[65] Irish WD, McCollum DA, Tesi RJ, Owen AB, Brennan DC, Bailly JE (2003). Nomogram for predicting the likelihood of delayed graft function in adult cadaveric renal transplant recipients. J Am Soc Nephrol.

[66] Lechevallier E, Dussol B, Luccioni A, Thirion X, Vacher-Copomat H, Jaber K (1998). Posttransplantation acute tubular necrosis: risk factors and implications for graft survival. Am J Kidney Dis.

[67] Cavaille-Coll M, Bala S, Velidedeoglu E, Hernandez A, Archdeacon P, Gonzalez G (2013). Summary of FDA workshop on ischemia reperfusion injury in kidney transplantation. Am J Transplant.

[68] Sharif A, Borrows R (2013). Delayed graft function after kidney transplantation: the clinical perspective. Am J Kidney Dis.

[69] Legendre C, Canaud G, Martinez F (2014). Factors influencing long-term outcome after kidney transplantation. Transpl Int.

[70] Yarlagadda SG, Coca SG, Formica RN, Poggio ED, Parikh CR (2009). Association between delayed graft function and allograft and patient survival: a systematic review and meta-analysis. Nephrol Dial Transplant.

[71] Butala NM, Reese PP, Doshi MD, Parikh CR (2013). Is delayed graft function causally associated with long-term outcomes after kidney transplantation? Instrumental variable analysis. Transplantation.

[72] Brennan DC, Daller JA, Lake KD, Cibrik D, Del Castillo D, Thymoglobulin Induction Study Group (2006). Rabbit antithymocyte globulin versus basiliximab in renal transplantation. N Engl J Med.

[73] Jeldres C, Cardinal H, Duclos A, Shariat SF, Suardi N, Capitanio U (2009). Prediction of delayed graft function after renal transplantation. Can Urol Assoc J.

[74] Irish WD, Ilsley JN, Schnitzler MA, Feng S, Brennan DC (2010). A risk prediction model for delayed graft function in the current era of deceased donor renal transplantation. Am J Transplant.

[75] Gourishankar S, Grebe SO, Mueller TF (2013). Prediction of kidney graft failure using clinical scoring tools. Clin Transplant.

[76] Rodrigo E, Minambres E, Ruiz JC, Ballesteros A, Pinera C, Quintanar J (2012). Prediction of delayed graft function by means of a novel web-based calculator: a single-center experience. Am J Transplant.

[77] Kaisar MO, Johnson DW (2006). Validation of a nomogram for predicting the likelihood of delayed graft function in Australian adult deceased donor renal transplant recipients. Nephrology (Carlton).

[78] Chapal M, Le Borgne F, Legendre C, Kreis H, Mourad G, Garrigue V (2014). A useful scoring system for the prediction and management of delayed graft function following kidney transplantation from cadaveric donors. Kidney Int.

[79] Morrissey PE (2007). Applicability of a nomogram to predict DGF. Transplantation.

[80] Moers C, Smits JM, Maathuis MH, Treckmann J, van Gelder F, Napieralski BP (2009). Machine perfusion or cold storage in deceased-donor kidney transplantation. N Engl J Med.

[81] O'Callaghan JM, Morgan RD, Knight SR, Morris PJ (2013). Systematic review and meta-analysis of hypothermic machine perfusion versus static cold storage of kidney allografts on transplant outcomes. Br J Surg.

[82] Jiao B, Liu S, Liu H, Cheng D, Cheng Y, Liu Y (2013). Hypothermic machine perfusion reduces delayed graft function and improves one-year graft survival of kidneys from expanded criteria donors: a meta-analysis. PLoS ONE.

[83] Deng R, Gu G, Wang D, Tai Q, Wu L, Ju W (2013). Machine perfusion versus cold storage of kidneys derived from donation after cardiac death: a meta-analysis. PLoS ONE.

[84] Bond M, Pitt M, Akoh J, Moxham T, Hoyle M, Anderson R (2009). The effectiveness and cost-effectiveness of methods of storing donated kidneys from deceased donors: a systematic review and economic model. Health Technol Assess.

[85] Canadian Institutes of Health Research. Guide to Knowledge Translation Planning at CIHR:Integrated and End-of-Grant Approaches. 2012; Available from: http://www.cihr-irsc.gc.ca/e/documents/kt_lm_ktplan-en.pdf.

[86] Canadian Institutes of health Research. More about knowledge translation at CIHR*.* 2014; Available from: http://www.cihr-irsc.gc.ca/e/39033.html.

[87] Bellomo R, Cass A, Cole L, Finfer S, Gallagher M, Lo S (2009). Intensity of continuous renal-replacement therapy in critically ill patients. N Engl J Med.

[88] Palevsky PM, Zhang JH, O'Connor TZ, Chertow GM, Crowley ST, Choudhury D (2008). Intensity of renal support in critically ill patients with acute kidney injury. N Engl J Med.

[89] Disease K (2012). Improving Global Outcomes (KDIGO) Acute Kidney Injury Work Group. KDIGO Clinical Practice Guideline for Acute Kidney Injury Kidney Int Suppl.

[90] Harel Z, Wald R, Bargman JM, Mamdani M, Etchells E, Garg AX (2013). Nephrologist follow-up improves all-cause mortality of severe acute kidney injury survivors. Kidney Int.

[91] Kidney Disease: Improving Global Outcomes (KDIGO) CKD Work Group (2013). KDIGO 2012 Clinical Practice Guideline for the Evaluation and Management of Chronic Kidney Disease. Kidney Int Suppl.

[92] Dasgupta K, Quinn RR, Zarnke KB, Rabi DM, Ravani P, Daskalopoulou SS (2014). The 2014 Canadian Hypertension Education Program Recommendations for Blood Pressure Measurement, Diagnosis, Assessment of Risk, Prevention, and Treatment of Hypertension. Can J of Cardiol.

[93] Anderson TJ, Grégoire J, Hegele RA, Couture P, Mancini GBJ, McPherson R (2012). Update of the Canadian Cardiovascular Society Guidelines for the Diagnosis and Treatment of Dyslipidemia for the Prevention of Cardiovascular Disease in the Adult. Can J of Cardiol.

[94] Disease K, Improving Global Outcomes (KDIGO) Lipid Work Group (2013). KDIGO Clinical Practice Guideline for Lipid Management in Chronic Kidney Disease. Kidney Int Suppl.

[95] Canadian Diabetes Association Clinical Practice Guidelines Expert Committee (2013). Canadian Diabetes Association 2013 Clinical Practice Guidelines for the Prevention and Management of Diabetes in Canada. Can J Diabetes.

[96] Chawla LS, Eggers PW, Star RA, Kimmel PL (2014). Acute kidney injury and chronic kidney disease as interconnected syndromes. N Engl J Med.

[97] Siew ED, Peterson JF, Eden SK, Hung AM, Speroff T, Ikizler TA (2012). Outpatient nephrology referral rates after acute kidney injury. J Am Soc Nephrol.

[98] Arora P, Obrador GT, Ruthazer R, Kausz AT, Meyer KB, Jenuleson CS (1999). Prevalence, predictors, and consequences of late nephrology referral at a tertiary care center. J Am Soc Nephrol.

[99] Hughes SA, Mendelssohn JG, Tobe SW, McFarlane PA, Mendelssohn DC (2013). Factors associated with suboptimal initiation of dialysis despite early nephrologist referral. Nephrol Dial Transplant.

[100] Mendelssohn DC, Curtis B, Yeates K, Langlois S, MacRae JM, Semeniuk LM (2011). Suboptimal initiation of dialysis with and without early referral to a nephrologist. Nephrol Dial Transplant.

[101] Smart NA, Titus TT (2011). Outcomes of Early versus Late Nephrology Referral in Chronic Kidney Disease: A Systematic Review. Am J Med.

[102] Allen AS, Forman JP, Orav EJ, Bates DW, Denker BM, Sequist TD (2011). Primary care management of chronic kidney disease. J Gen Intern Med.

[103] Avorn J, Bohn RL, Levy E, Levin R, Owen WF, Winkelmayer WC (2002). Nephrologist care and mortality in patients with chronic renal insufficiency. Arch Intern Med.

[104] Coleman EA, Smith JD, Raha D, Min SJ (2005). Posthospital medication discrepancies: prevalence and contributing factors. Arch Intern Med.

[105] Tam VC, Knowles SR, Cornish PL, Fine N, Marchesano R, Etchells EE (2005). Frequency, type and clinical importance of medication history errors at admission to hospital: a systematic review. CMAJ.

[106] Bell CM, Brener SS, Gunraj N, Huo C, Bierman AS, Scales DC (2011). Association of ICU or hospital admission with unintentional discontinuation of medications for chronic diseases. JAMA.

[107] Wu VC, Wu CH, Huang TM, Wang CY, Lai CF, Shiao CC (2014). Long-term risk of coronary events after AKI. J Am Soc Nephrol.

[108] Chawla LS, Amdur RL, Shaw AD, Faselis C, Palant CE, Kimmel PL (2014). Association between AKI and long-term renal and cardiovascular outcomes in United States veterans. Clin J Am Soc Nephrol.

[109] Bucaloiu ID, Kirchner HL, Norfolk ER, Hartle JE, Perkins RM (2012). Increased risk of death and de novo chronic kidney disease following reversible acute kidney injury. Kidney Int.

[110] Campbell M (2000). Framework for design and evaluation of complex interventions to improve health. BMJ.

[111] Polichnowski AJ, Lan R, Geng H, Griffin KA, Venkatachalam MA, Bidani AK (2014). Severe Renal Mass Reduction Impairs Recovery and Promotes Fibrosis after AKI. J Am Soc Nephrol.

[112] Fassett RG, Venuthurupalli SK, Gobe GC, Coombes JS, Cooper MA, Hoy WE (2011). Biomarkers in chronic kidney disease: a review. Kidney Int.

[113] Chawla LS, Amdur RL, Amodeo S, Kimmel PL, Palant CE (2011). The severity of acute kidney injury predicts progression to chronic kidney disease. Kidney Int.

[114] Harel Z, Bell CM, Dixon SN, McArthur E, James MT, Garg AX (2014). Predictors of progression to chronic dialysis in survivors of severe acute kidney injury: a competing risk study. BMC Nephrol.

[115] Levinson W, Rothman AI, Phillipson E (2006). Creative professional activity: an additional platform for promotion of faculty. Acad Med.

